# Effects of Strigolactones on NLRP3 Activation, Nitrosative Stress, and Antioxidant Mox Phenotype: In Vitro and In Silico Evidence

**DOI:** 10.1021/acsbiomedchemau.3c00063

**Published:** 2024-02-20

**Authors:** Gizem Antika, Zeynep Özlem Cinar, Serhat Dönmez, Esma Seçen, Mehmet Özbil, Cristina Prandi, Tugba Boyunegmez Tumer

**Affiliations:** † Graduate Program of Molecular Biology and Genetics, School of Graduate Studies, Canakkale Onsekiz Mart University, Canakkale 17020, Turkey; ‡ Graduate Program of Molecular Medicine, Universitätsklinikum Jena, Friedrich-Schiller-Universität Jena, Jena 07740, Germany; § Institute of Biotechnology, Gebze Technical University, Kocaeli 41400, Turkey; ∥ Department of Chemistry, University of Turin, Turin 10125, Italy; ⊥ Department of Molecular Biology and Genetics, Faculty of Science, Canakkale Onsekiz Mart University, Canakkale 17020, Turkey

**Keywords:** SL analogues, NLRP3 inflammasome, microglial activation, Mox phenotype, Nitrosative stress, SIM-A9 cells

## Abstract

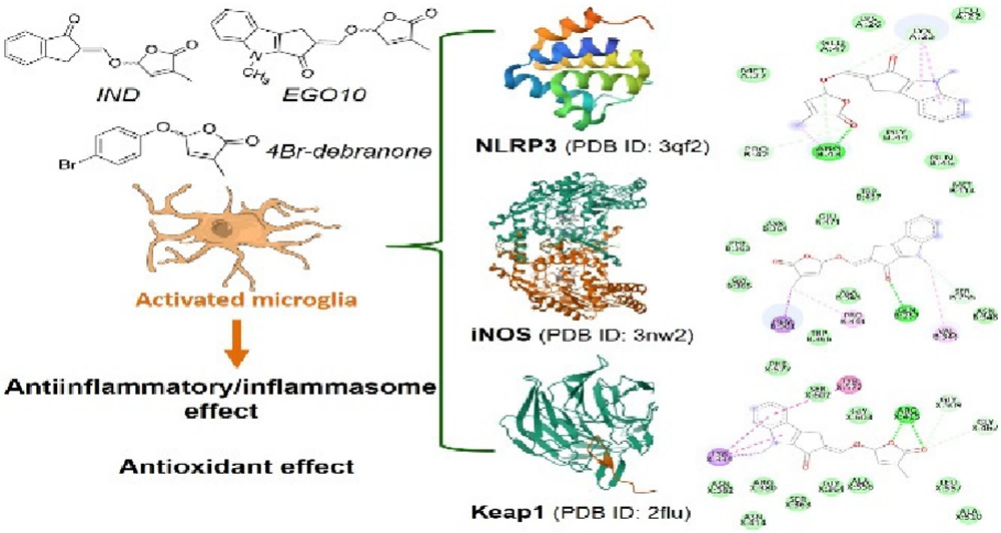

Phytohormones have significant roles in redox metabolism, inflammatory responses, and cellular survival mechanisms within the microenvironment of the mammalian brain. Herein, we identified the mammalian molecular targets of three representative strigolactone (SL) analogues structurally derived from apocarotenoids and the functional equivalent of plant hormones. All tested SL analogues have an inhibitory effect on NLRP3 inflammasome-mediated IL-1β release in murine microglial cells. However, IND and EGO10 became prominent among them due to their high potency at low micromolar doses. All SL analogues dose-dependently suppressed the release and expression of proinflammatory factors. For EGO10 and IND, IC_50_ values for iNOS-associated NO secretion were as low as 1.72 and 1.02 μM, respectively. In silico analyses revealed that (*S*)-EGO10 interacted with iNOS, NLRP3, and Keap1 ligands with the highest binding affinities among all stereoisomeric SL analogues. Although all compounds were effective in microglial Mox phenotype polarization, 4-Br-debranone exhibited a differential pattern for upregulating Nrf2-driven downstream enzymes.

Strigolactones (SLs) are one of the phytohormone classes, and at least 25 natural SL molecules have been isolated and characterized so far.^1,2^ Because of their complex structures, synthesizing and isolating these compounds from their natural sources involve challenging procedures. For this reason, researchers have attempted to synthesize a variety of SL molecules with simpler structures that retain biological activities. SLs containing ABC-rings are called canonical strigolactones, others that show simpler structures lacking one or two rings of the ABC system are generally termed noncanonical SL-like compounds.^3−5^ In this context, SL molecules investigated in the current work involve both canonical (EGO10) and noncanonical (indanone-derived SL-IND and 4-Br-debranone) structures (see Table 1).

**Table 1:**
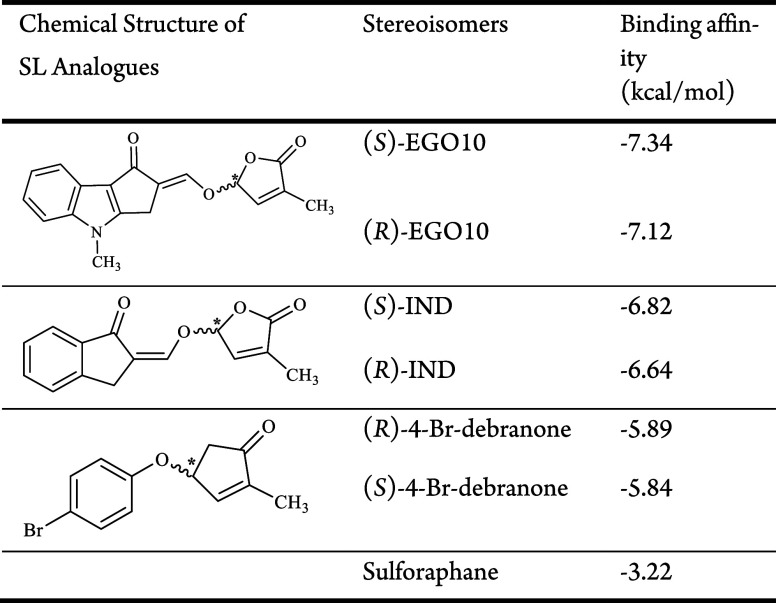
Binding Affinities of SL Analogues to NLRP3 Protein^a^

a The symbol (*) represented in the chemical structures of three SL analogues shows the chiral carbon atoms.

Recently, we have elucidated that GR24, a synthetic SL analogue, suppressed neuroinflammatory determinants in lipopolysaccharide (LPS) stressed microglial cells by mediating NF-κB, Nrf2, and PPARγ pathways. More in detail, GR24 attenuated LPS-induced permeability in the BBB endothelial cells by upregulating the expression of tight junction genes.^2^ Afterward, in our previous work, the two bioactiphores IND and EGO10 became prominent due to their potent inhibitory activities on glioblastoma cell proliferation by inducing apoptosis and G1 cell cycle arrest at very low concentrations.^6^ Additionally, ADME analyses of these compounds demonstrated that they have MW less than 500 g/mol, feasible polar surface area, lipophilicity, and a BBB partition coefficient for cellular and BBB permeability.^6^ In the current work, EGO10, IND, and 4-Br-debranone came to the forefront due to their potent role in the inhibition of nitrosative stress, NLR family pyrin domain containing 3 (NLRP3) inflammasome-mediated IL-1β release, and switching of microglial phenotype by in vitro and in silico analyses.

It is well-known that at micromolar concentration NO confers nitrosative stress, which is neurotoxic to the brain.^2^ iNOS-derived NO is released from astrocytes and microglial cells to the microenvironment of the brain and reaches toxic levels under the inflammatory stimulus, aggravating the conditions by creating a detrimental loop between other inflammatory insults. According to the MTT assay (Figure S1), in microglia cells, EGO10 and IND were not cytotoxic (>80% cellular viability) up to 10 μM. 4-Br-debranone did not demonstrate any toxicity up to 50 μM. In the following with these safety doses, all SL analogues significantly and dose-dependently attenuated the NO secretion level in LPS-induced SIM-A9 cells (Figure 1). The half-maximal inhibitory concentrations (IC_50_) for EGO10 and IND were detected as 1.72 and 1.02 μM, respectively, which are lower than IC_50_ assessed for the selective and irreversible inhibitor of iNOS, 1400W (2.2 μM).^2^

**Figure 1:**
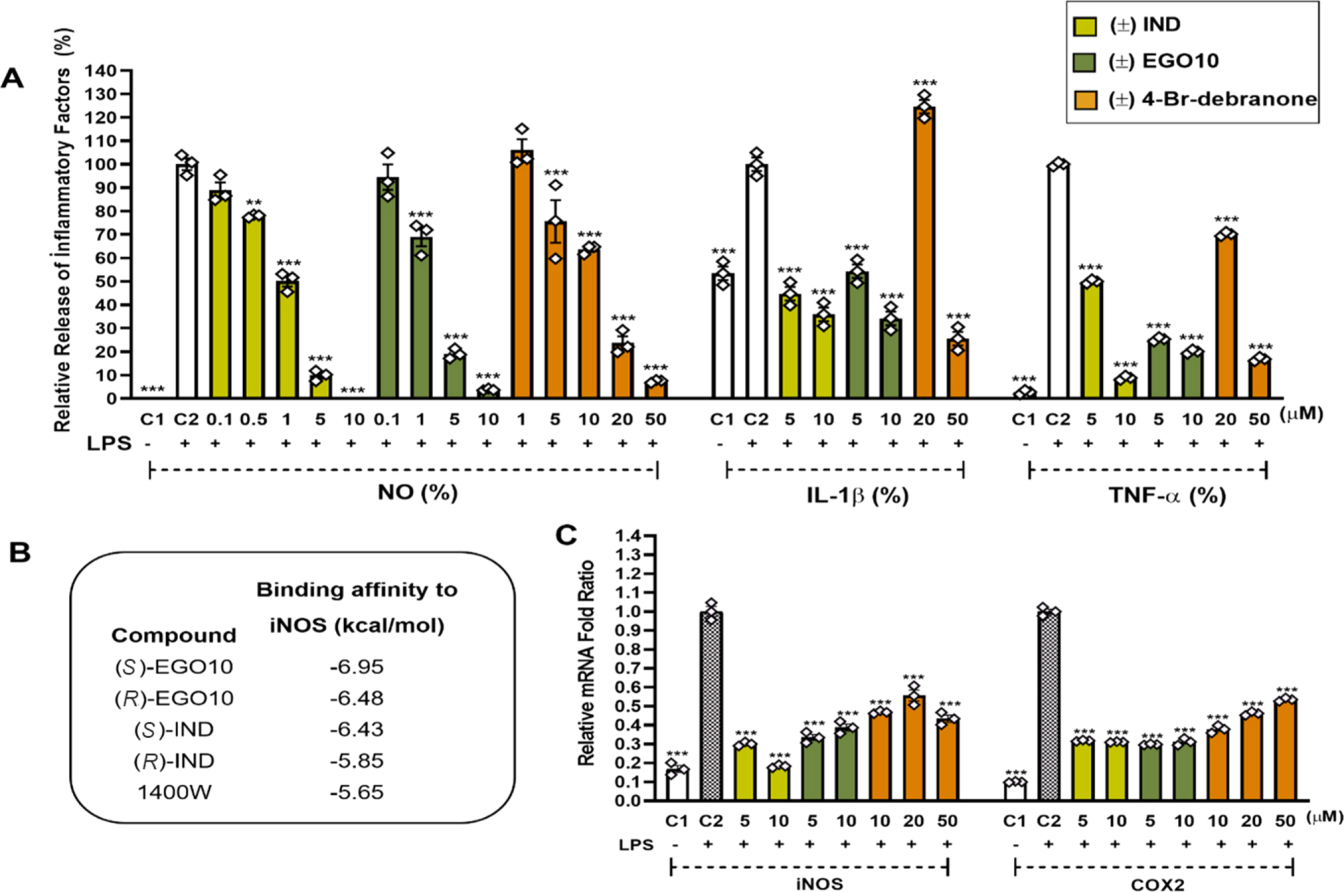
Effects of SL analogues on M1 proinflammatory phenotype markers. (A) The relative levels of NO, IL-1β, and TNF-α release from LPS-induced SIM-A9 cells with or without SL treatment for 24 h. (B) Binding affinities of ± EGO10, ± IND, and 1400W to iNOS. (C) Effects of SL analogues on the mRNA expression levels of iNOS and COX-2 in LPS-induced SIM-A9 cells for 12 h. C1: treatment with only DMSO (vehicle), C2: only LPS. The symbols (◇) represent the triplicate individual analysis, and the bars represent the mean ± SEM of three independent experiments. **p < 0.005 and ***p < 0.001.

Molecular docking studies were carried out to reveal interactions between SL analogues and iNOS. Accordingly, (*S*)-EGO10 provided the highest binding affinity (−6.95 kcal/mol). Its stereoisomer (*R*)-EGO10 and (*S*)-IND provided similar binding affinities of −6.48 and −6.43 kcal/mol, respectively. (*R*)-IND bound to the protein with −5.85 kcal/mol. All of these ligands provided higher binding affinity than the reference molecule 1400W (Figure 1B). Both enantiomers of EGO10 and IND interacted with residues Gln 257, Val 346, and Hem 901 found in the active site of the iNOS protein (Figure S2). Therefore, ± EGO10 and ± IND may be candidates as iNOS inhibitors; however, studies using pure enzyme systems and selectivity studies considering other NOS isoforms are necessary.

5 and 10 μM doses of both EGO10 and IND were also found to be highly effective in suppressing LPS-induced IL-1β and TNF-α release when microglial secretomes were analyzed. EGO10 decreased the level of LPS-induced IL-1β release by 46% (5 μM) and 66% (10 μM), and IND attenuated its level by 55 and 64%, dose-dependently, at the same concentrations (Figure 1A). In the case of TNF-α, the suppression level reached 80% for EGO10 and 91% for IND at 10 μM as compared to the control (LPS). In terms of 4-Br-debranone at only 50 μM, more than 70% suppression levels were reached for both LPS-induced IL-1β and TNF-α releases. All compounds significantly downregulated the mRNA expressions of proinflammatory enzymes (iNOS and COX-2) and mediators, except TNF-α (Figures 1C and S3). Although the compounds were found to be very active in inhibiting LPS-induced TNF-α release, the same trend was not exhibited at the mRNA expression level. It has been known that TNF synthesis is not entirely associated with DNA-to-mRNA transcription, and nearly, all regulations of TNF synthesis appear to occur at the post-transcriptional level.^7^ The gene expression of COX-2 decreased by 68% with the treatments of both IND and EGO10, and 62% decreased with 4-Br-debranone at 10 μM concentration (Figure 1C).

Activation of the NLRP3 inflammasome is a crucial mechanism in the acceleration of neuroinflammation. The multiprotein complex is triggered by microbial (LPS) or endogenous molecules as the first signal that induces the NF-κB pathway. A second signal such as ATP, pore-forming toxins (nigericin), or viral RNA triggers the assembling of the NLRP3 inflammasome complex, eventually leading to proteolytic cleavage of pro-IL-1β into the active IL-1β.^8^

A recent in vitro study showed that 5 μM sulforaphane inhibited the NLRP3 inflammasome activation in LPS and ATP-induced N9 microglial cells.^9^ Moreover, NLRP3 inhibition in microglial cells with a small molecule MCC950 resulted in reduced Aβ pathology, suggesting that reduced NLRP3 activity may be ameliorative in AD treatment.^10^

In the current study, SIM-A9 microglia cells were subjected to a priming step for 24 h with LPS followed by 40 min of 1 mM of ATP induction, which is an in vitro protocol for the activation of NLRP3 inflammasome through a P2X7 purinergic receptor on the plasma membrane (Figure 2A).^9,11,12^ The ATP treatment following LPS presented a remarkable increase in the level of IL-1β secretion by 63% compared to LPS-treated SIM-A9 cells. As illustrated in Figure 2B, EGO10 at 10 μM almost completely inhibited the NLRP3-dependent IL-1β release in LPS+ATP-induced SIM-A9 cells. The IL-1β secretion level was significantly and dose-dependently suppressed by 5 and 10 μM of IND by 27 and 47%; 20 and 50 μM of 4-Br-debranone resulted in 74 and 84% decrease as compared to the LPS+ATP-induced control. Moreover, while 5 μM sulforaphane (positive control) suppressed the IL-1β level by 87%, 5 μM EGO10 led to an 80% decrease in ATP+LPS-induced SIM-A9 cells. The in vitro NLRP3 inflammasome activation model was also conducted by the treatment of 10 μM of nigericin (NIG) as another activator, which is a potassium ionophore that directly enters the cell and causes the efflux of intracellular K^+^ ions.^13^ In NIG+LPS-treated SIM-A9 cells, the NLRP3-induced IL-1β release was almost completely suppressed by the treatment of IND and EGO10 at 5 μM, while 20 μM of 4-Br-debranone led to a 16% decrease as compared to the control (Figure S4).

**Figure 2:**
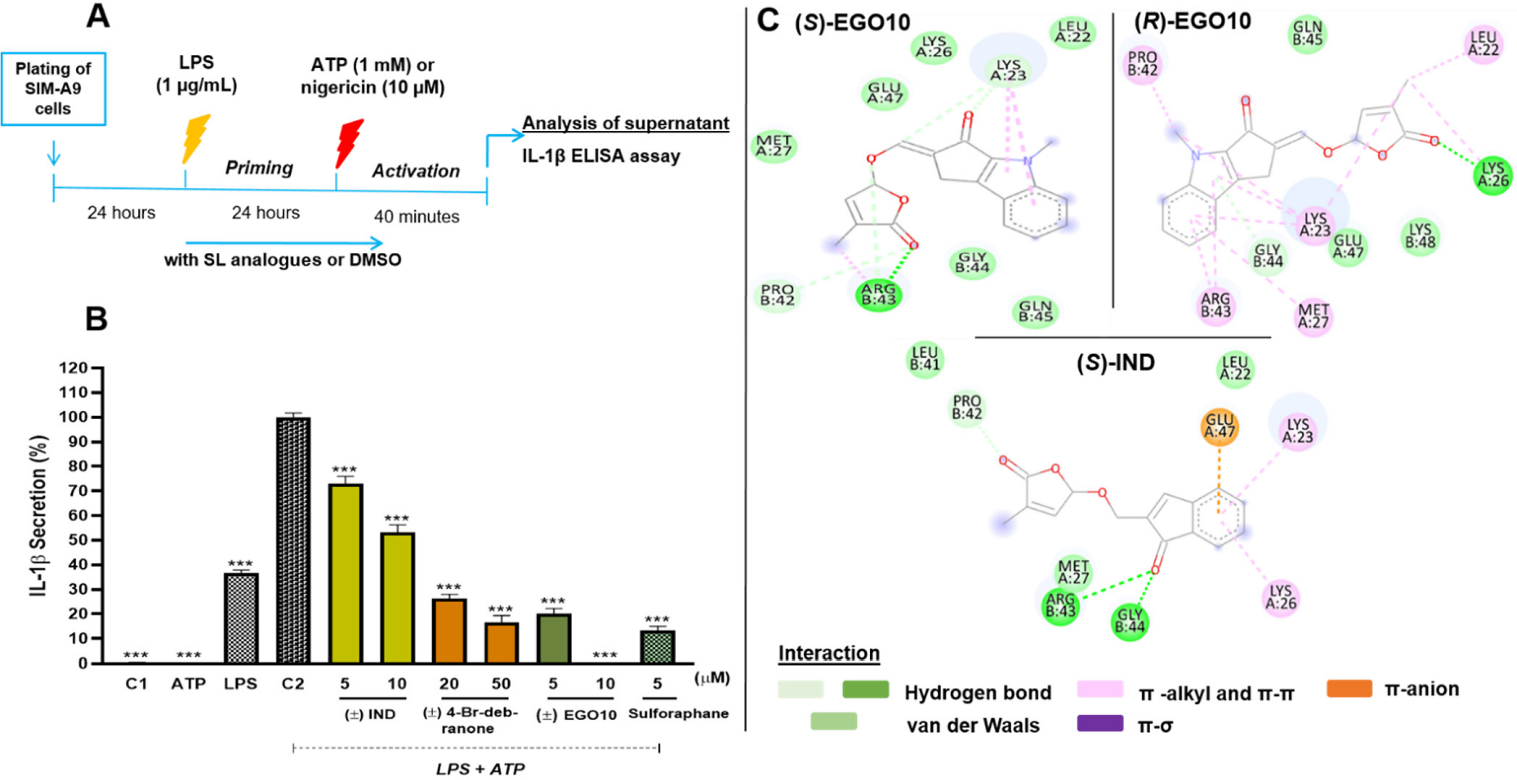
Action of SLs on the NLRP3 inflammasome activation model. (A) In vitro protocol for NLRP3 inflammasome activation and (B) inhibitory effects of SLs on NLRP3 inflammasome-mediated IL-1β release in SIM-A9 cells. (C) Interacting residues of NLRP3 with (*S*)-EGO10, (*R*)-EGO10, and (*S*)-IND. C1: treatment with only DMSO (vehicle), ATP: only ATP, LPS: only LPS, C2: LPS+ATP-treated control group. The symbol (◇) represents the triplicate individual analysis, and the bars represent the mean ± SEM of three independent experiments. ***p < 0.001.

Each stereoisomer of ± EGO10, ± IND, and ± 4-Br-debranone was examined for their potential to bind with the active sites of NLRP3 (PDB ID: 3qf2). According to binding affinities obtained from molecular docking simulations (*S*)-EGO10 bound to the protein with the highest affinity, −7.34 kcal/mol, which is followed by (*R*)-EGO10 with slightly lower binding affinity (−7.12 kcal/mol) (Table 1). Although other SL analogues resulted in significantly lower binding affinities, they bound to the protein much more strongly than sulforaphane, the reference molecule. All the SL analogues interacted with the Leu 22, Lys 23, Lys 26, Pro 42, and Arg 43 amino acids in the binding site of the NLRP3 through different types of interactions. For instance, (*S*)-EGO10 generated five hydrogen bonds, two π-alkyl, one alkyl-alkyl interaction, and six van der Waals interactions with the binding site of the NLRP3. On the contrary, (*R*)-EGO10 yielded two hydrogen bonds, five π-alkyl interactions; two alkyl-alkyl interactions, and three van der Waals interactions with the same amino acid residues. These variations clearly emphasized the significance of ligand stereochemistry (Table 1, and Figure 2C). Atomic interactions of the other four ligands with NLRP3 are represented in Figure S5.

Microglia cells possess an ability to switch their phenotypes, i.e., polarization/activation states under different conditions. While M1 is the predominant phenotype in the case of inflammation and is associated with various neurodegenerative disorders, M2 is responsible for the resolution of inflammation and damage repair.^14,15^ The gene expression levels of M2 phenotype markers including Arg1 and CD206 decreased after LPS stimulation by 0.6- and 0.5-fold in SIM-A9 cells compared to nontreated control, respectively (Figure S6). However, IND increased the gene expression level of Arg1 by 1.8-fold at 5 μM and the level of CD206 gene by 1.7-fold at 10 μM concentration in LPS-induced SIM-A9 cells. As a subtype macrophage phenotype, the Mox phenotype was represented by an obvious upregulation of Nrf2-mediated expression of redox-regulatory genes including HO-1, Srxn-1, and Gclc.^16^ In this study, the Mox phenotype switching effects of SLs on the inflammation model of microglial cells were identified for the first time. As seen in Figure 3A, 4-Br-debranone at 20 μM induced all Nrf2 regulated enzymes between 1.5- and 3.8-fold; at 50 μM, the level of induction reached 2.0–15.4-fold. The most significant inductions were detected for the NQO1 and HO-1 enzymes. For EGO10 and IND at 10 μM, the fold increases elevated quite high values (up to 6.5 fold especially for NQO1, Figure 3B).

**Figure 3:**
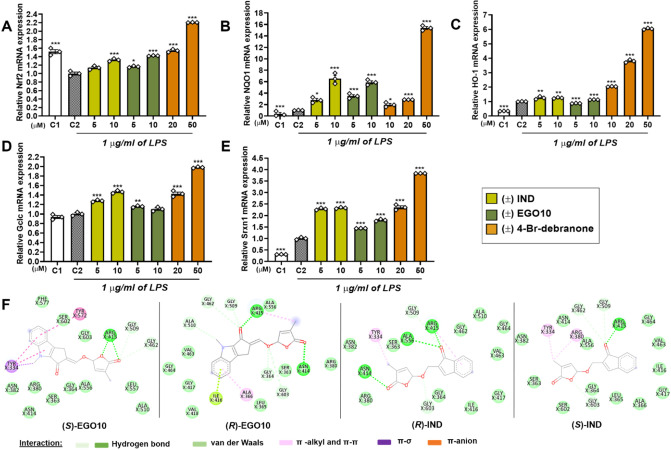
Promoting effects of SL analogues on (A) Nrf2, (B) NQO1, (C) HO-1, (D) Gclc, and (E) Srxn1 mRNA expression levels after 12 h in SIM-A9 cells. (F) Interacting residues of Keap1 with (*S*)-EGO10, (*R*)-EGO10, (*R*)-IND, and (*S*)-IND. C1: including only DMSO (vehicle); C2: only treatment with LPS. The symbols represent the triplicate individual analysis, and the bars represent the mean ± SEM of three independent experiments. *p < 0.02, **p < 0.005, and ***p < 0.001.

Kelch-like ECH-associated protein 1 (Keap1) regulates the Nrf2 signaling pathway promoting Nrf2 degradation through ubiquitination.^17^ According to the molecular docking studies performed between these SL analogues and the Kelch domain of Keap1 (PDB ID: 2flu), (*S*)*-*EGO10, (*R*)*-*EGO10, and (*R*)*-*IND showed higher binding affinities at the Nrf2 binding site of the Keap1, −8.22, −7.66, and −7.13 kcal/mol, respectively. EGCG is also used as a positive control due to its structural similarity to the SL analogues. The compound bound to the protein with a binding affinity of −4.25. (Table 2).

**Table 2:** Binding Affinity of SL Analogues, Sulforaphane, and EGCG through Keap1

Compounds	Binding affinity (kcal/mol)
(*S*)-EGO10	–8.22
(*R*)-EGO10	–7.66
(*R*)-IND	–7.13
(*S*)-IND	–6.73
(*S*)-4-Br-debranone	–6.56
(*R*)-4-Br-debranone	–6.29
Sulforaphane	–4.00
EGCG	–4.25

Even though both EGO10 stereoisomers formed similar interactions with the Keap1 binding pocket, the 0.56 kcal/mol binding affinity difference between *-R* and *-S* configuration was probably caused by the increased number of the π-π and π-σ interactions between the tricyclic lactone group of (*S*)*-*EGO10 and NLRP3. The binding affinity difference between (*R*)-IND and (*S*)-IND might be due to the presence of one extra hydrogen bond between Asn 414 and the lactone carbonyl oxygen of (*R*)-IND (Figure 3F). Atomic interactions for the ± 4-Br-debranone were supplemented in Figure S7. Overall, all SL analogues provided significantly higher binding affinities than reference molecules; sulforaphane and EGCG (Table 2).

In conclusion, EGO10 and IND, both compounds, showed very prominent activity in inhibiting NLRP3 inflammasome-mediated IL-1β release at low μM doses. In the case of in silico studies, (*S*)-EGO10 showed the strongest binding affinity to iNOS, NLRP3, and Keap1 proteins. Based on the common fact that inflammation is the unifying source for tumor development and progression, herein current findings enforced the hypothesis in our previous report^6^ suggesting that EGO10 and IND may present two promising pharmacophores for the development of novel multipotent antiglioma agents. In our next phase, we aim to optimize novel biomimetic derivatives based on the chemical structures of EGO10 and IND, employing a fragment-based pharmacophore design approach that focuses on the structural motifs. Subsequently, in silico-optimized SL-like pharmacophores were developed, synthesized, and screened for further preclinical and clinical investigations.

## Supporting Information Available

Experimental methods; effects of these SLs on the cell viability of SIM-A9 microglia cells; LPS+NIG induced IL-1β release; gene expression levels of IL-1β, TNF-α, Arg1, and CD206 on LPS-induced SIM-A9 cells; interactions of SL analogues with iNOS, NLRP3, and Keap1 (PDF)
