# Supplementary material for: Effects of Strigolactones on NLRP3 Activation, Nitrosative Stress, and Antioxidant Mox Phenotype: In Vitro and In Silico Evidence
Source: ACS Bio Med Chem Au. 2024 Feb 20;4(3):131–6. doi: 10.1021/acsbiomedchemau.3c00063 (PMC11191569; doi:10.1021/acsbiomedchemau.3c00063)
Supplement: Supplementary file 1 — bg3c00063_si_001.pdf [file bg3c00063_si_001.pdf]

SUPPORTING INFORMATION

Effects of Strigolactones on NLRP3 Activation, Nitrosative Stress, and Antioxidant Mox Phenotype: In Vitro and In Silico Evidences

Gizem Antika[1], Zeynep Özlem Cinar[1], Serhat Dönmez[1], Esma Seçen[2], Mehmet Özbil[3], Cristina Prandi[4], Tugba Boyunegmez Tumer[5*]

[1] Graduate Program of Molecular Biology and Genetics, School of Graduate Studies, Canakkale Onsekiz Mart University, Canakkale 17020, Turkey

[2] Graduate Program of Molecular Medicine, Universitätsklinikum Jena, Friedrich-Schiller-Universität Jena, Jena 07740, Germany

[3] Institute of Biotechnology, Gebze Technical University, Kocaeli 41400, Turkey

[4] Department of Chemistry, University of Turin, Turin 10125, Italy

[5] Department of Molecular Biology and Genetics, Faculty of Science, Canakkale Onsekiz Mart University, Canakkale 17020, Turkey.

Figure S1: Effects of SL analogs on cell viability of SIM-A9 microglia cells.........................S2

Figure S2: Interacting residues of iNOS with (*S*)-EGO10, (*R*)-EGO10, (*S*)-IND, (*R*)-IND, and 1400W.........................S2

Figure S3: Effects of SL analogs on the mRNA expression levels of IL-1β and TNF-α in LPS-induced SIM-A9 microglia cells for 12 hours.........................S3

Figure S4: Inhibitory effects of SLs on NLRP3 inflammasome-mediated IL-1β release after stimulation with LPS and 10 μM of nigericin.........................S3

Figure S5: Binding site and interacting residues of NLRP3 with (*R*)-IND, (*R*)-GR24, (*R*)-4-Br-debranone, (*S*)-4-Br-debranone, and sulforaphane.........................S4

Figure S6: Effects of SL analogs on mRNA expression levels of M2 phenotype markers including (A) Arg1 and (B) CD206 genes in LPS-induced SIM-A9 cells.........................S5

Figure S7: Interacting residues of Keap1 with (±) 4-Br-debranone, sulforaphane, and EGCG.........................S6

Experimental Methods.........................S7

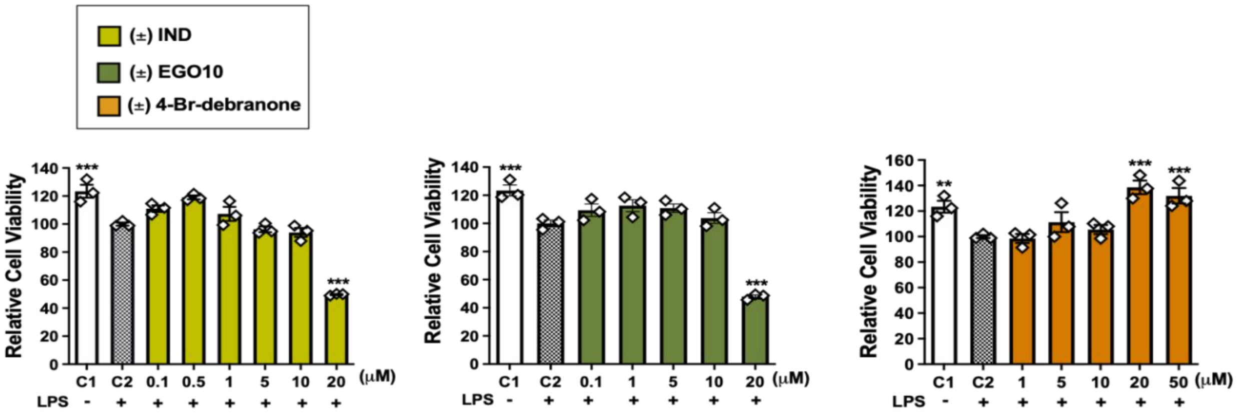

Figure S1. Effects of SL analogs on cell viability of SIM-A9 microglia cells. C1: including only DMSO (vehicle), C2: only treatment with 1 μg/mL of LPS. The symbol (◇) represents the triplicate individual analysis and the bars represent the mean ± SEM of three independent experiments (n=3 per group). **p* < 0.02, and ****p* < 0.001 vs. LPS-treated alone group (C2).

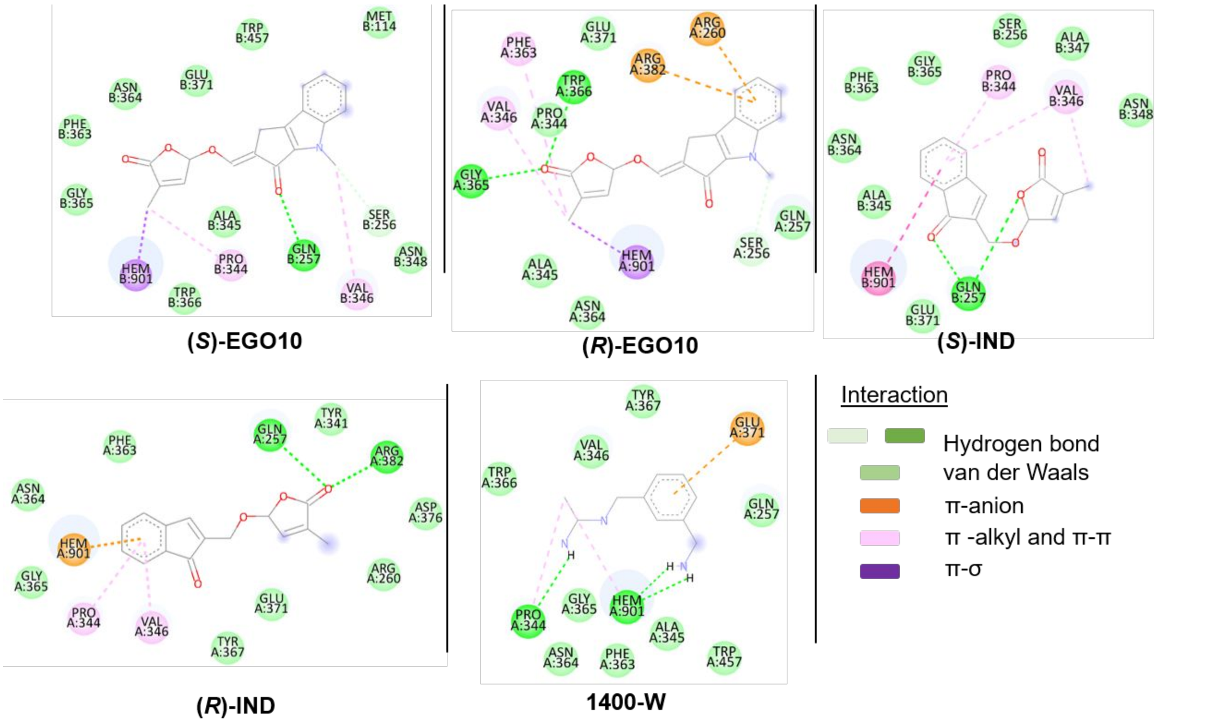

Figure S2. Interacting residues of iNOS with (*S*)-EGO10, (*R*)-EGO10, (*S*)-IND, (*R*)-IND, and 1400W.

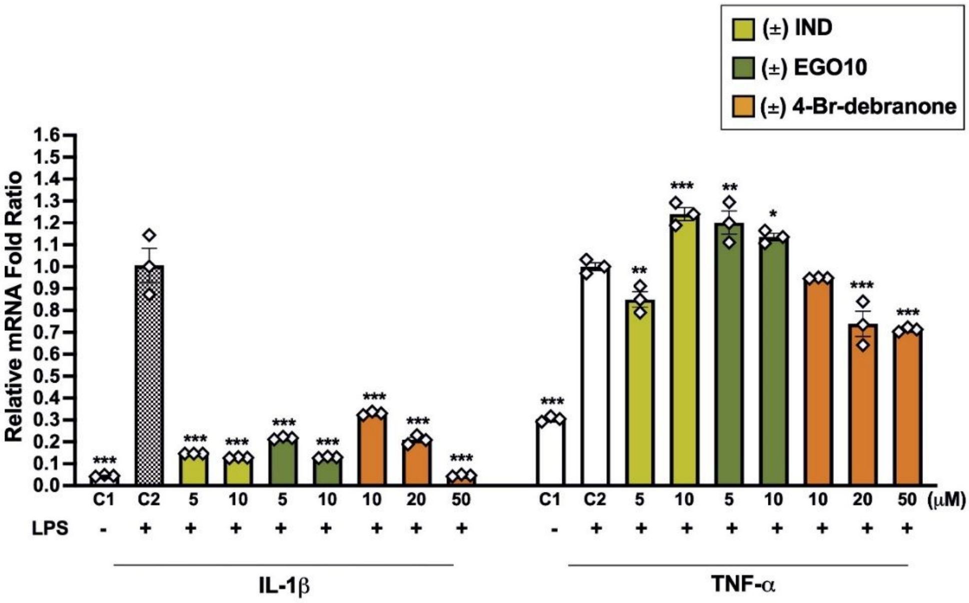

Figure S3. Effects of SL analogs on the mRNA expression levels of IL-1β and TNF-α in LPS-induced SIM-A9 microglia cells for 12 hours. C1: including only DMSO (vehicle), C2: only treatment with 1 μg/mL of LPS. The symbols (◇) represent the triplicate individual analysis and the bars represent the mean ± SEM of three independent experiments (n=3 per group). *p < 0.02, **p < 0.005, and ***p < 0.001 vs. LPS-treated alone group (C2).

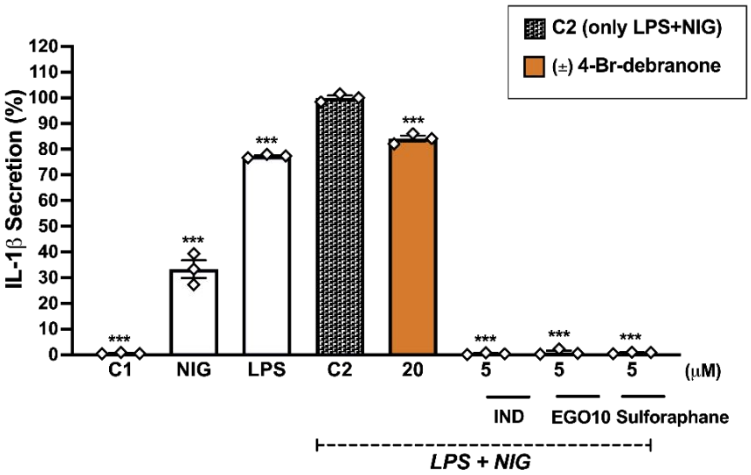

Figure S4. Inhibitory effects of SLs on NLRP3 inflammasome-mediated IL-1β release after stimulation with LPS and 10 μM of nigericin. C1: including only DMSO (vehicle), NIG: only treatment with 10 μM of nigericin, LPS: only treatment with 1 μg/mL of LPS, C2: LPS+NIG-treated control group. The symbol (◇) represents the triplicate individual analysis and the bars

represent the mean ± SEM of three independent experiments (n=3 per group). ***$p < 0.0001$ vs. LPS+ATP-treated alone group (C2).

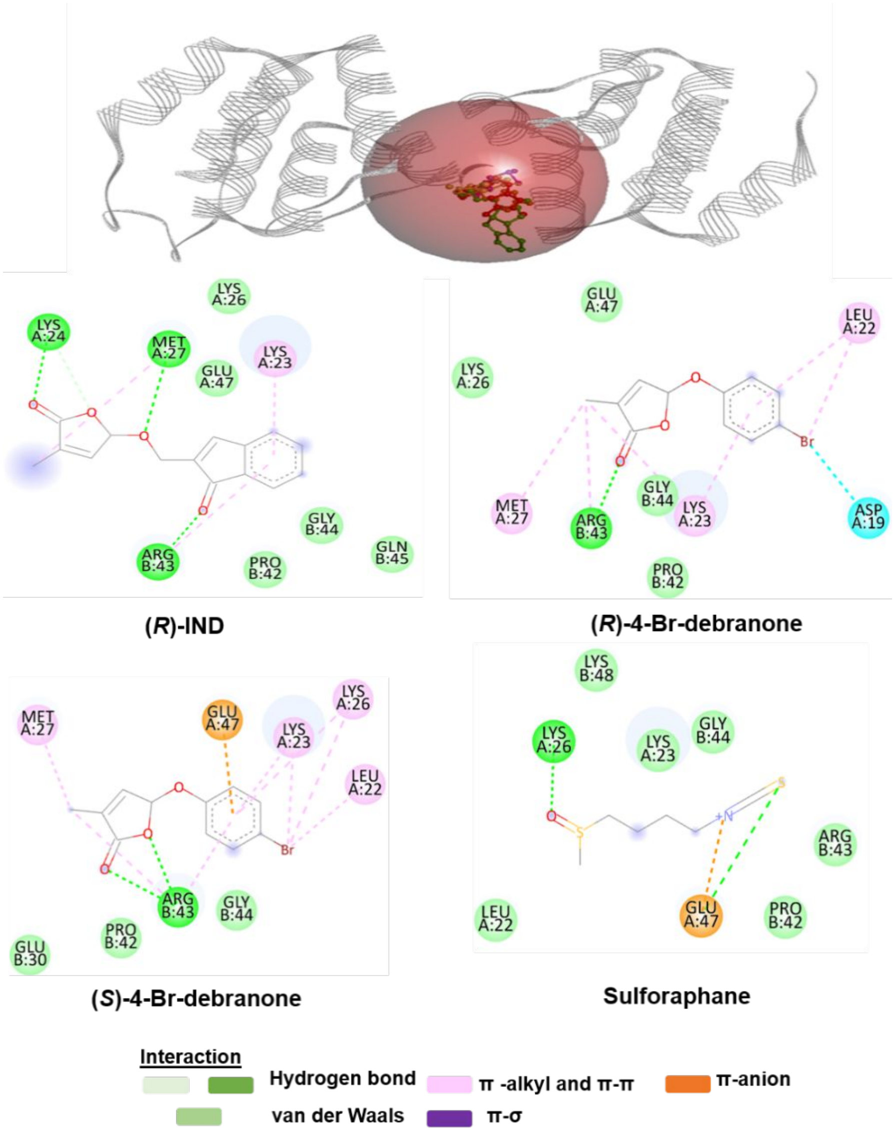

Figure S5. Binding site and interacting residues of NLRP3 with (*R*)-IND, (*R*)-4-Br-debranone, (*S*)-4-Br-debranone, and sulforaphane.

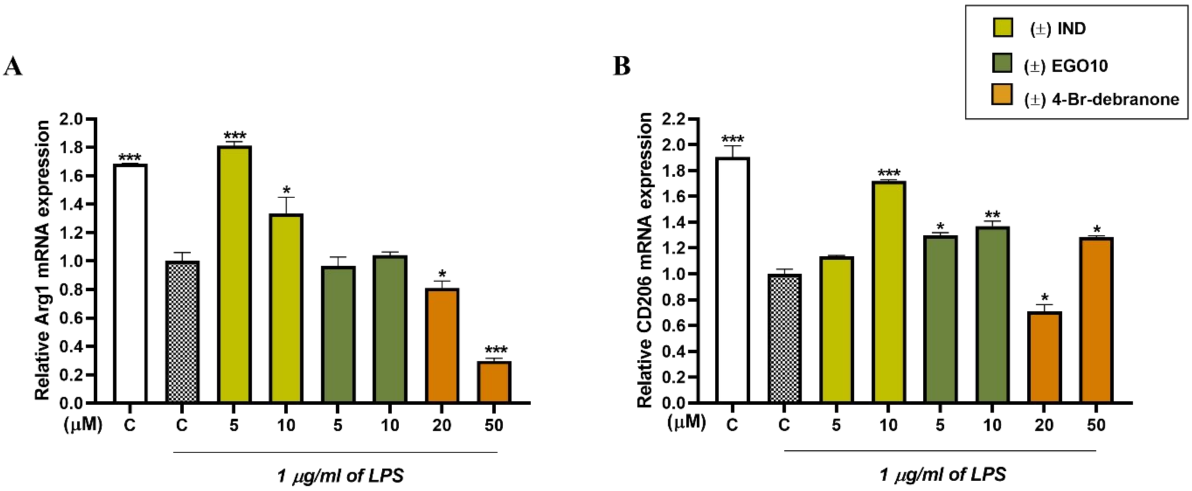

Figure S6. Effects of SL analogs on mRNA expression levels of M2 phenotype markers including (A) Arg1 and (B) CD206 genes in LPS-induced SIM-A9 cells. C1: including only DMSO (vehicle), C2: only treatment with 1 µg/mL of LPS. The symbols (◇) represent the triplicate individual analysis and the bars represent the mean ± SEM of three independent experiments (n=3 per group). **p* < 0.02, ***p* < 0.005, and ***p < 0.001 vs. LPS-treated alone group (C2).

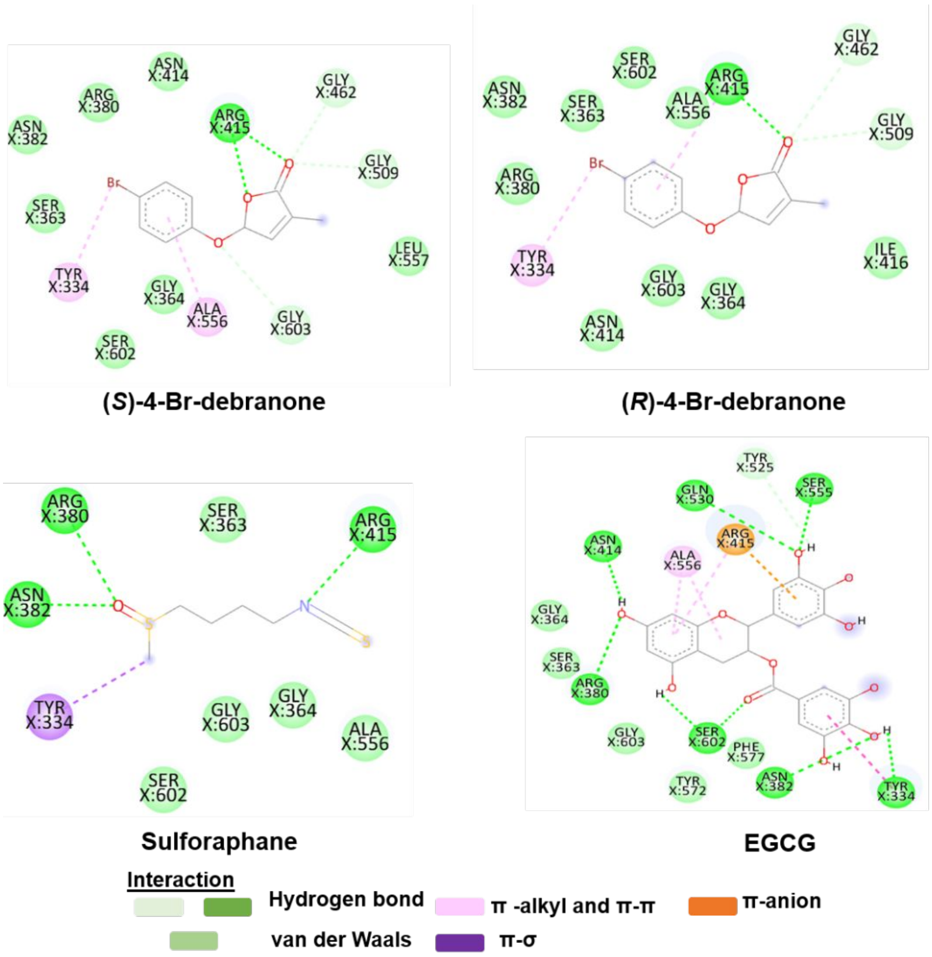

Figure S7. Interacting residues of Keap1 with (±) 4-Br-debranone, sulforaphane, and EGCG.

Experimental Methods

Cell culture and treatments

SIM-A9 mouse microglial cell line (ATCC CRL-3265) was purchased from American Type Culture Collection (ATCC, Manassas, VA, USA). SIM-A9 microglia cells were grown in DMEM/F12 (Gibco, Paisley, UK) medium including 10% fetal bovine serum (FBS), 5% horse serum, and 1% penicillin-streptomycin in an incubator comprising 5% CO_2 and 75% humidity at 37 °C. Inflammatory in vitro model in SIM-A9 cells was established by the treatment with 1 µg/ml of LPS (Lipopolysaccharides from *E. Coli* O55:B5, Cat. No. L6529) for 12 hours (gene expression analysis) or 24 hours (cell viability, nitric oxide (NO), and ELISA assays).

MTT cell viability assay

Cells were seeded into 24-well plates at a concentration of $4x10^5$ cells/well and incubated for 24 hours. After the cells were treated and incubated for 24 hours under appropriate conditions, the medium of each sample was aspirated gently. The 20 µL MTT at 0.1 mg/mL concentration was simultaneously added to each well and incubated for two hours at 37 ° C, 5% CO_2, and 75% humidity conditions. The medium was aspirated, and the formazan formed by living cells was dissolved with 200 µL of DMSO for 10 minutes. Cell survival rates were quantified at 570 nm and 620 nm (background) using a Tecan Infinite M200 PRO microplate reader.

NO release measurement and IC_{50} determination

NO release levels were determined by analyzing the media collected from the inflammation model of SIM-A9 microglia cells according to the Griess method as previously described.[1] Different doses of SL analogs were administered to SIM-A9 cells together with 1 µg/mL of LPS and incubated for 24 hours. After 24 hours, 100 µL medium for each sample and sodium nitrite ($NaNO_2$) standards were mixed with Griess reagent at equal amounts and incubated in a 96-well plate for 10 minutes at room temperature and in a dark environment. The absorbance of color that formed after incubation was quantified at 520 nm using the microplate reader. The calculated NO concentrations were normalized by cell viability percentages. IC_{50} and ANOVA analyses were performed with the GraphPad Prism 8 program.

TNF-α and IL-1β ELISA assays

First of all, SIM-A9 cells were seeded at a density of $4x10^5$ cells/well and incubated for 24 hours. Subsequently, media was exchanged with DMEM media without serum, and selected doses of SL analogs with LPS were applied to the SIM-A9 cells. After 24 hours of incubation, the collected media were analyzed using Invitrogen mouse IL-1β and TNF-α ELISA kits (ThermoFisher Invitrogen, Cat. No. BMS6002 and BMS607-3) according to the protocols provided by the manufacturer. The concentrations of IL-1β and TNF-α secreted into the medium were normalized to the total concentration of cellular protein measured by the BCA assay. IL-1β ELISA assay was also carried out for the samples of LPS+ATP/nigericin-induced SIM-A9 cells which were treated with the aforementioned doses of SL analogs.

NLRP3 inflammasome activation model in SIM-A9 cells

SIM-A9 cells were treated firstly with LPS in the presence or absence of SL analogs at selected concentrations and 5 μM of sulforaphane (Cayman Chemical, USA, item no 10496) as a positive control or DMSO as vehicle control[2,3] for 24 hours. After LPS priming, the treatment process followed with 1 mM of adenosine 5′-triphosphate (ATP) (Sigma Aldrich, Cat. No. A6419) or 10 μM of nigericin (Tocris Bioscience, Cat No. 4312) for 40 minutes to switch on the NLRP3 inflammasome mechanism.[4,5] The supernatants were collected to evaluate the effects of SL analogs against NLRP3-mediated IL-1β release in microglia cells by ELISA assay.

Gene expression analysis

SIM-A9 cells were treated with certain doses of SL analogs and LPS. After 12 hours, the total RNA of each sample was collected from the cell pellet by the NORGEN Total RNA Purification Plus kit according to the protocols provided by the manufacturer. The cDNA synthesis was actualized using the ABI-High-Capacity cDNA reverse transcriptase kit, according to the manufacturer's instructions. Relative gene expression levels of samples were analyzed by Real-Time Quantitative Polymerase Chain Reaction (RT-qPCR) that was performed by using cDNAs and TaqMan probes specific for iNOS, IL-1β, COX-2, TNF-α, NQO1, HO-1, Gclc, Srxn1, and Nrf2 genes.

Molecular docking simulations

Molecular docking simulations were performed in AutoDock 4.2.6 software.[6] SL analogs were designed using ChemDraw Structure software (RRID: SCR 016768). 3D structures of 1400W (PubChem CID: 1433), sulforaphane (PubChem CID: 5350), and EGCG (PubChem CID: 65064) molecules were downloaded from PubChem.[7] All ligands were subjected to energy minimization in YASARA Structure and UCSF Chimera softwares utilizing the Amber 03 force field.[7–9] 3D structure of NLRP3 (PDB ID: 3qf2),[10] KEAP1 (PDB ID: 2flu), and iNOS (PDB ID: 3nw2)[11] were downloaded from the RCSB Protein data bank (rcsb.org).[12] Water molecules, inhibitors, and ions were removed from the structure of proteins. Polar hydrogens and Kollman charges were added to the atoms of the proteins and Gastiger charges were added to ligands prior to docking studies. Grid boxes were generated according to the active site of the proteins. The dimensions for boxes were 40 x 40 x 40 Å for NLRP3, 40 x 50 x 60 Å for KEAP1, and 82 x 80 x 126 Å for iNOS. Fifty Lamarckian genetic algorithm runs with 300 population sizes were carried out for docking studies. The best docking pose for each complex was chosen according to the docking affinity score (more negative value meaning better binding). Amino acid interactions were visualized in Discovery Studio 2021 Client software (BIOVIA, Dassault Systeèmes, Discovery Studio Client, San Diego: Dassault Systeèmes, 2021).

Statistical analysis

All data were analyzed statistically using One Way ANOVA with Dunnett's post hoc test and a 95% confidence interval by GraphPad Prism 8 software (GraphPad Software, Inc., San Diego, CA) (*$p < 0.02$, **$p < 0.005$, ***$p < 0.001$). The mean of each column was compared with the mean of a control column, which is represented as C2 and corresponds to the SIM-A9 cells induced by LPS or LPS+ATP/NIG depending on the evaluation of the inflammatory or NLRP3 inflammasome activation conditions. All results are presented as the individual values of three separate experiments and bars plotted for the mean ± SEM of each group.

REFERENCES

(1) Kapche, D. W. F. G.; Lekane, N. M.; Kulabas, S. S.; Ipek, H.; Tok, T. T.; Ngadjui, B. T.; Demirtas, I.; Tumer, T. B. Aryl Benzofuran Derivatives from the Stem Bark of Calpocalyx Dinklagei Attenuate Inflammation. *Phytochemistry* **2017**, *141*, 70–79. DOI:

10.1016/J.PHYTOCHEM.2017.05.007.

(2) Lee, J.; Ahn, H.; Hong, E. J.; An, B. S.; Jeung, E. B.; Lee, G. S. Sulforaphane Attenuates Activation of NLRP3 and NLRC4 Inflammasomes but Not AIM2 Inflammasome. *Cell. Immunol.* **2016**, *306–307*, 53–60. DOI: 10.1016/J.CELLIMM.2016.07.007.

(3) Tufekci, K. U.; Ercan, I.; Isci, K. B.; Olcum, M.; Tastan, B.; Gonul, C. P.; Genc, K.; Genc, S. Sulforaphane Inhibits NLRP3 Inflammasome Activation in Microglia through Nrf2-Mediated MiRNA Alteration. *Immunol. Lett.* **2021**, *233*, 20–30. DOI: 10.1016/j.imlet.2021.03.004.

(4) Hseu, Y. C.; Tseng, Y. F.; Pandey, S.; Shrestha, S.; Lin, K. Y.; Lin, C. W.; Lee, C. C.; Huang, S. T.; Yang, H. L. Coenzyme Q0Inhibits NLRP3 Inflammasome Activation through Mitophagy Induction in LPS/ATP-Stimulated Macrophages. *Oxid. Med. Cell. Longev.* **2022**, *2022*. DOI: 10.1155/2022/4266214.

(5) Qiu, J.; Chen, Y.; Zhuo, J.; Zhang, L.; Liu, J.; Wang, B.; Sun, D.; Yu, S.; Lou, H. Urolithin A Promotes Mitophagy and Suppresses NLRP3 Inflammasome Activation in Lipopolysaccharide-Induced BV2 Microglial Cells and MPTP-Induced Parkinson's Disease Model. *Neuropharmacology* **2022**, *207*, 108963. DOI: 10.1016/j.neuropharm.2022.108963.

(6) Morris, G. M.; Ruth, H.; Lindstrom, W.; Sanner, M. F.; Belew, R. K.; Goodsell, D. S.; Olson, A. J. Software News and Updates AutoDock4 and AutoDockTools4: Automated Docking with Selective Receptor Flexibility. *J. Comput. Chem.* **2009**, *30* (16), 2785–2791. DOI: 10.1002/jcc.21256.

(7) Kim, S.; Chen, J.; Cheng, T.; Gindulyte, A.; He, J.; He, S.; Li, Q.; Shoemaker, B. A.; Thiessen, P. A.; Yu, B.; Zaslavsky, L.; Zhang, J.; Bolton, E. E. PubChem in 2021: New Data Content and Improved Web Interfaces. *Nucleic Acids Res.* **2021**, *49* (D1), D1388–D1395. DOI: 10.1093/NAR/GKAA971.

(8) Duan, Y.; Wu, C.; Chowdhury, S.; Lee, M. C.; Xiong, G.; Zhang, W.; Yang, R.; Cieplak, P.; Luo, R.; Lee, T.; Caldwell, J.; Wang, J.; Kollman, P. A Point-Charge Force Field for Molecular Mechanics Simulations of Proteins Based on Condensed-Phase Quantum Mechanical Calculations. *J. Comput. Chem.* **2003**, *24* (16), 1999–2012. DOI:

10.1002/jcc.10349.

(9) Krieger, E.; Darden, T.; Nabuurs, S. B.; Finkelstein, A.; Vriend, G. Making Optimal Use of Empirical Energy Functions: Force-Field Parameterization in Crystal Space. *Proteins Struct. Funct. Genet.* **2004**, *57* (4), 678–683. DOI: 10.1002/prot.20251.

(10) Bae, J. Y.; Park, H. H. Crystal Structure of NALP3 Protein Pyrin Domain (PYD) and Its Implications in Inflammasome Assembly. *J. Biol. Chem.* **2011**, *286* (45), 39528–39536. DOI: 10.1074/jbc.M111.278812.

(11) Lo, S. C.; Li, X.; Henzl, M. T.; Beamer, L. J.; Hannink, M. Structure of the Keap1:Nrf2 Interface Provides Mechanistic Insight into Nrf2 Signaling. *EMBO J.* **2006**, *25* (15), 3605–3617. DOI: 10.1038/sj.emboj.7601243.

(12) Berman, H. M.; Westbrook, J.; Feng, Z.; Gilliland, G.; Bhat, T. N.; Weissig, H.; Shindyalov, I. N.; Bourne, P. E. The Protein Data Bank. *Nucleic Acids Res.* **2000**, *28* (1), 235–242. DOI: 10.1093/NAR/28.1.235.
